# Predicting Transvaginal Surgical Mesh Exposure Outcomes Using an Integrated Dataset of Blood Cytokine Levels and Medical Record Data: Machine Learning Approach

**DOI:** 10.2196/59631

**Published:** 2025-05-01

**Authors:** Mihyun Lim Waugh, Tyler Mills, Nicholas Boltin, Lauren Wolf, Patti Parker, Ronnie Horner, Thomas L Wheeler II, Richard L Goodwin, Melissa A Moss

**Affiliations:** 1 Department of Biomedical Engineering, University of South Carolina, 301 Main St, Rm 2C02, Columbia, SC, 29208-4101, United States, 1 8646336181; 2 University of South Carolina School of Medicine, Columbia, SC, United States; 3 Prisma Health, Greenville, SC, United States; 4 Department of Health Services Research and Administration, University of Nebraska Medical Center, Omaha, NE, United States; 5 Department of Obstetrics and Gynecology, Spartanburg Regional Healthcare, Spartanburg, SC, United States

**Keywords:** cytokines, inflammatory response, medical record, pelvic organ prolapse, polypropylene mesh, supervised machine learning models, polypropylene, mesh surgery, surgical outcome, cost-efficiency, risk factor, efficacy, health care data, female, informed decision-making, patient care, digital health

## Abstract

**Background:**

Transvaginal insertion of polypropylene mesh was extensively used in surgical procedures to treat pelvic organ prolapse (POP) due to its cost-efficiency and durability. However, studies have reported a high rate of complications, including mesh exposure through the vaginal wall. Developing predictive models via supervised machine learning holds promise in identifying risk factors associated with such complications, thereby facilitating better informed surgical decisions. Previous studies have demonstrated the efficacy of anticipating medical outcomes by employing supervised machine learning approaches that integrate patient health care data with laboratory findings. However, such an approach has not been adopted within the realm of POP mesh surgery.

**Objective:**

We examined the efficacy of supervised machine learning to predict mesh exposure following transvaginal POP surgery using 3 different datasets: (1) patient medical record data, (2) biomaterial-induced blood cytokine levels, and (3) the integration of both.

**Methods:**

Blood samples and medical record data were collected from 20 female patients who had prior surgical intervention for POP using transvaginal polypropylene mesh. Of these subjects, 10 had experienced mesh exposure through the vaginal wall following surgery, and 10 had not. Standardized medical record data, including vital signs, previous diagnoses, and social history, were acquired from patient records. In addition, cytokine levels in patient blood samples incubated with sterile polypropylene mesh were measured via multiplex assay. Datasets were created with patient medical record data alone, blood cytokine levels alone, and the integration of both data. The data were split into 70% and 30% for training and testing sets, respectively, for machine learning models that predicted the presence or absence of postsurgical mesh exposure.

**Results:**

Upon training the models with patient medical record data, systolic blood pressure, pulse pressure, and a history of alcohol usage emerged as the most significant factors for predicting mesh exposure. Conversely, when the models were trained solely on blood cytokine levels, interleukin (IL)-1β and IL-12 p40 stood out as the most influential cytokines in predicting mesh exposure. Using the combined dataset, new factors emerged as the primary predictors of mesh exposure: IL-8, tumor necrosis factor-α, and the presence of hemorrhoids. Remarkably, models trained on the integrated dataset demonstrated superior predictive capabilities with a prediction accuracy as high as 94%, surpassing the predictive performance of individual datasets.

**Conclusions:**

Supervised machine learning models demonstrated improved prediction accuracy when trained using a composite dataset that combined patient medical record data and biomaterial-induced blood cytokine levels, surpassing the performance of models trained with either dataset in isolation. This result underscores the advantage of integrating health care data with blood biomarkers, presenting a promising avenue for predicting surgical outcomes in not only POP mesh procedures but also other surgeries involving biomaterials. Such an approach has the potential to enhance informed decision-making for both patients and surgeons, ultimately elevating the standard of patient care.

## Introduction

Patients experiencing difficulty from symptoms of pelvic organ prolapse (POP) are prescribed nonsurgical option as a first-line treatment, including pelvic floor physical therapy to strengthen the muscles supporting the pelvic organs and the use of pessaries that are inserted into the vagina to provide structural support and alleviate symptoms. Lifestyle modifications are also suggested, such as weight management and avoidance of heavy lifting, to reduce symptoms. However, conservative treatments often fail to provide adequate relief, leading many patients to pursue surgical interventions. Notably, patients who undergo surgical treatments are more likely to report complete satisfaction 1 year after the procedure compared to those who opt for nonsurgical options [1]. The choice of surgical approach depends largely on the type of prolapse. Among these, transvaginal mesh surgery was once a widely used treatment when reinforcement of the pelvic floor structures was deemed necessary to treat severe or recurrent prolapse, though its use was later restricted following safety concerns raised by the Food and Drug Administration. This paper focuses on transvaginal mesh surgery to explore strategies for identifying patient populations that are more likely to experience favorable surgical outcomes, with the aim of minimizing the number of patients who face less successful results.

Surgical treatment via transvaginal mesh implantation uses polypropylene mesh due to its low cost, nontoxicity, and mechanical resilience. Its superior tensile strength and elasticity compared to native pelvic tissue enabled it to withstand the pressure changes within the pelvic cavity [2]. Despite these advantageous properties, the usage of polypropylene mesh in transvaginal repair of POP has been prohibited in multiple countries due to postsurgical complications, notably mesh exposure through the vaginal wall [2,3]. In fact, studies have reported mesh exposure rates ranging from 4% to 12.3% in POP patients following surgery [4-6]. Conversely, however, nearly 90% of patients experience successful POP treatment via transvaginal mesh. Consequently, accurately identifying risk factors and developing personalized predictive models can greatly benefit both patients and surgeons in making informed surgical decisions.

Building a machine learning model to predict postsurgical mesh exposure holds considerable promise, as it offers a robust framework that harnesses large datasets, identifies relevant patterns, and provides quantitative predictions that can be used as clinical decision-making tools. In fact, supervised machine learning models have demonstrated efficacy in predicting various surgical outcomes, including patient survival, symptom improvement, and disease progression [7-9]. This efficacy is particularly evident when using datasets that integrate patient health care information and biological assessments. For instance, Jung et al [7] developed an individualized predictive model incorporating patient health care information from medical records as well as pre- and postoperative laboratory measurements (eg, total bilirubin and sodium levels) to anticipate graft failure in pediatric liver transplant surgery. Similarly, Chowdhury et al [8] applied biomarkers, such as mucus cytokines, along with patient demographics and clinical characteristics to machine learning algorithms to predict chronic rhinosinusitis improvement when treated with endoscopic sinus surgery. Kawakita et al [9] developed a model to predict delayed graft function with kidney transplantation surgery by integrating donor and recipient health care characteristics (eg, BMI, race, age, diabetes diagnosis) and clinical laboratory data (eg, blood urea nitrogen levels, serum glutamic pyruvic transaminase). Despite these successful examples of incorporating demographic, clinicopathological, therapeutic, and biomolecular data into personalized clinical decision-making tools aimed at predicting poor clinical outcomes, such methods have yet to gain acceptance in the current clinical practice of mesh surgery.

In this preliminary study, we explored the benefit of integrating distinct datasets when developing supervised machine learning models to predict mesh exposure in patients who have undergone POP repair surgery involving transvaginal insertion of polypropylene mesh. Models were trained with 3 different datasets: patient medical record data (such as demographics, social history, and medical history), biomaterial-induced blood cytokine levels, and the integration of both datasets. Models trained with these 3 different datasets were able to identify pertinent health risk factors as well as important predictors among the cytokines. Moreover, models trained with the integrated dataset relative to individual datasets demonstrated superior performance. These outcomes illustrate the potential of leveraging integrated datasets with machine learning models to accurately predict transvaginal mesh implantation outcomes and identify crucial risk factors associated with such outcomes. Such predictive models offer invaluable guidance to patients and surgeons. This approach holds potential to enhance the decision-making process for both transvaginal and other mesh surgeries, leading to improved patient care and better surgical outcomes.

## Methods

### Study Population

As described previously [10], recruited patients with surgical intervention for POP using transvaginal polypropylene mesh included both those with mesh exposure, a postsurgical complication where the implanted mesh protrudes through the vaginal wall, and those without. A cohort of 20 healthy, nonpregnant female patients, divided evenly between the 2 categories, was determined necessary for statistical observation of a difference in blood cytokine biomarkers with a 10% chance of error. Briefly, this statistical analysis used a 1-tailed *t* test based on a priori power analysis, a Cohen *d* of 1, and a conservative study power of 0.80.

Participants were selected retrospectively from a list of patients who had undergone surgical intervention for POP using polypropylene mesh at Prisma Health Greenville Memorial Hospital, as detailed in Waugh et al [10]. The patients were randomly identified and recruited via phone call until 10 patients with mesh exposure and 10 without were enrolled for this study. Participants with POP recurrence or those taking medications that could affect inflammatory responses were excluded from the study.

### Ethical Considerations

The study protocol was approved by the Institutional Review Board of Prisma Health (number 1984257). Informed consent was waived due to the retrospective nature of the data. All collected data were deidentified and stored in a secure, password-protected folder with restricted access. As this was a retrospective study with no subject participation, no compensation was provided.

### Patient Medical Record Data Collection

Medical record data were collected from 20 female patients who had experienced prior surgical intervention for POP using transvaginal polypropylene mesh, selected retrospectively and randomly as described earlier. The data categories include vital signs (systolic blood pressure [BP], diastolic BP, and pulse pressure), previous medical history (hypertension, diabetes, renal disease, hyperlipidemia, hemorrhoids, endometriosis, gastroesophageal reflux, and hysterectomy), social history (alcohol and tobacco usage, sexual activity, marital status, and education level), and other relevant health statistics (age at surgery, BMI, BMI change since surgery, POP stage diagnosis at surgery, and number of births). Mean and SD values were calculated for continuous variables (systolic and diastolic BP, pulse pressure, age at surgery, BMI, and BMI changes) along with the range of the variables (Table 1). Unpaired *t* tests were conducted on each variable to determine significance (*P*<.05).

**Table 1.** Demographics of the patients with pelvic organ prolapse (POP) study population (N=20 subjects).

Variables	Mesh exposure (n=10 subjects)	No mesh exposure (n=10 subjects)
Vital signs		
Systolic blood pressure		
Mean (SD)	127.4 (17.9)	123.3 (19.3)
Range	102‐162	98‐152
Diastolic blood pressure		
Mean (SD)	77.9 (11.3)	76.9 (10.7)
Range	58‐98	60‐92
Pulse pressure		
Mean (SD)	49.5 (21.3)	46.4 (11.9)
Range	24‐104	
Previous medical history, n (%)		
Hypertension	4 (40)	5 (50)
Diabetes	0 (0)	2 (20)
Renal disease	2 (20)	5 (50)
Hyperlipidemia	3 (30)	3 (30)
Hemorrhoids	3 (30)	2 (20)
Endometriosis	3 (30)	4 (40)
Gastroesophageal reflux	5 (50)	6 (60)
Hysterectomy	8 (80)	8 (80)
Social history, n (%)		
Alcohol	6 (60)	2 (20)
Tobacco	2 (20)	1 (10)
Sexual activity	9 (90)	5 (50)
Marital status		
Single or lives alone	0 (0)	1 (10)
Married	8 (80)	6 (60)
Separated or divorced	1 (10)	1 (10)
Widowed	1 (10)	2 (20)
Highest education level		
Primary	0 (0)	1 (10)
Secondary	2 (20)	2 (20)
College or higher	8 (80)	7 (70)
Other relevant health statistics		
Age at surgery (years)		
Mean (SD)	56.8 (1.2)	59.4 (8.0)
Range	43‐77	47‐75
BMI		
Mean (SD)	29.3 (7.0)	28.8 (6.5)
Range	19.3-42.3	21.5‐44.9
BMI change, mean (SD)	−1.6 (4.5)	−1.1 (3.5)
POP stage preoperation, n (%)		
1	1 (10)	1 (10)
2	4 (40)	2 (20)
3	4 (40)	7 (70)
4	1 (10)	0 (0)
Number of births, n (%)		
1‐2	6 (60)	5 (50)
3+	4 (40)	5 (50)

### Patient Blood Cytokine Measurements

Patient blood samples were collected from the same 20 female patients and incubated with sterile polypropylene mesh (2 cm × 2 cm), as detailed in Waugh et al [10]. Plasma layers collected following centrifugation (1500xg, 4 °C) of blood samples were analyzed via multiplex assay to quantify cytokine levels, as in Waugh et al [10]. These cytokines include interleukin (IL)-1α, IL-1β, IL-2, IL-4, IL-6, IL-8, IL-10, IL-12 p40, IL-12 p70, IL-17A, interferon-γ, tumor necrosis factor-α (TNF-α), and granulocyte-macrophage colony-stimulating factor. In total, 3 independent measurements were observed to detect the cytokine levels, with each sample evaluated in duplicate.

### Data Analysis

Raw datasets, including blood cytokine levels and various medical record data, were analyzed using the statistical programming language R (RStudio, Inc). The imported data contained 60 observations (20 subjects x 3 independent cytokine measurements) and 35 total variable fields (21 medical record variables+13 cytokines+1 target variable). The target variable of exposure referred to the presence or absence of surgical mesh exposure through the vaginal wall following POP surgery. Univariate and multivariate analysis was implemented to explore the dataset, including identifying missing values, analyzing outliers, and standardizing categorical variables.

Supervised machine learning models were created using the caret package (version 6.0‐90) in the R programming language. The 4 models trained were decision tree (DT), logistic regression (LR), Naïve Bayes (NB), and artificial neural network (ANN). This approach focused on three different datasets: (1) patient medical record data, (2) blood cytokine levels following incubation with surgical mesh, and (3) an integrated dataset of both medical record data and blood cytokine levels. Prior to creating the predictive models, the original data were split using an industry standard of 70% for training and 30% for testing. Each group contained an equal distribution of subjects who did or did not experience postsurgical mesh exposure through the vaginal wall, the prediction target for each model. Each model was trained using the 70% (42/60) subset and a cross-validation training control. A 10-fold cross-validation with 25% (15/60) left out replicated 3 times was used on each model to avoid bias and overfitting. From this, training accuracies are reported. Additional testing was performed for the prediction accuracy of each model using the 30% (18/60) test data. Prediction accuracies are reported along with sensitivity and specificity for the prediction of subjects to experience postsurgical mesh exposure. After computing predictive statistics for each dataset, analysis using the varImp function was conducted to identify which variables contributed most to the predictive outcome within each model. This analysis allowed for visualization of individual variable importance regarding each predictive model.

## Results

### Descriptive Analysis of Patient Medical Record Data

Health care data of patients with POP were collected from electronic medical records and analyzed in Table 1. The cohort included 20 patients with POP, among whom were the 10 that experienced vaginal mesh exposure and 10 that did not. Overall, no significant differences (*P*<.05) in systolic and diastolic BP, pulse pressure, age at surgery, BMI, and BMI changes were noted between patients with or without mesh exposure. Higher numbers of patients with no mesh exposure had previous medical diagnoses of diabetes and renal diseases. In contrast, higher numbers of patients with mesh exposure reported social histories of alcohol usage as well as sexual activity.

### Predictive Analysis

**Figure 1.**
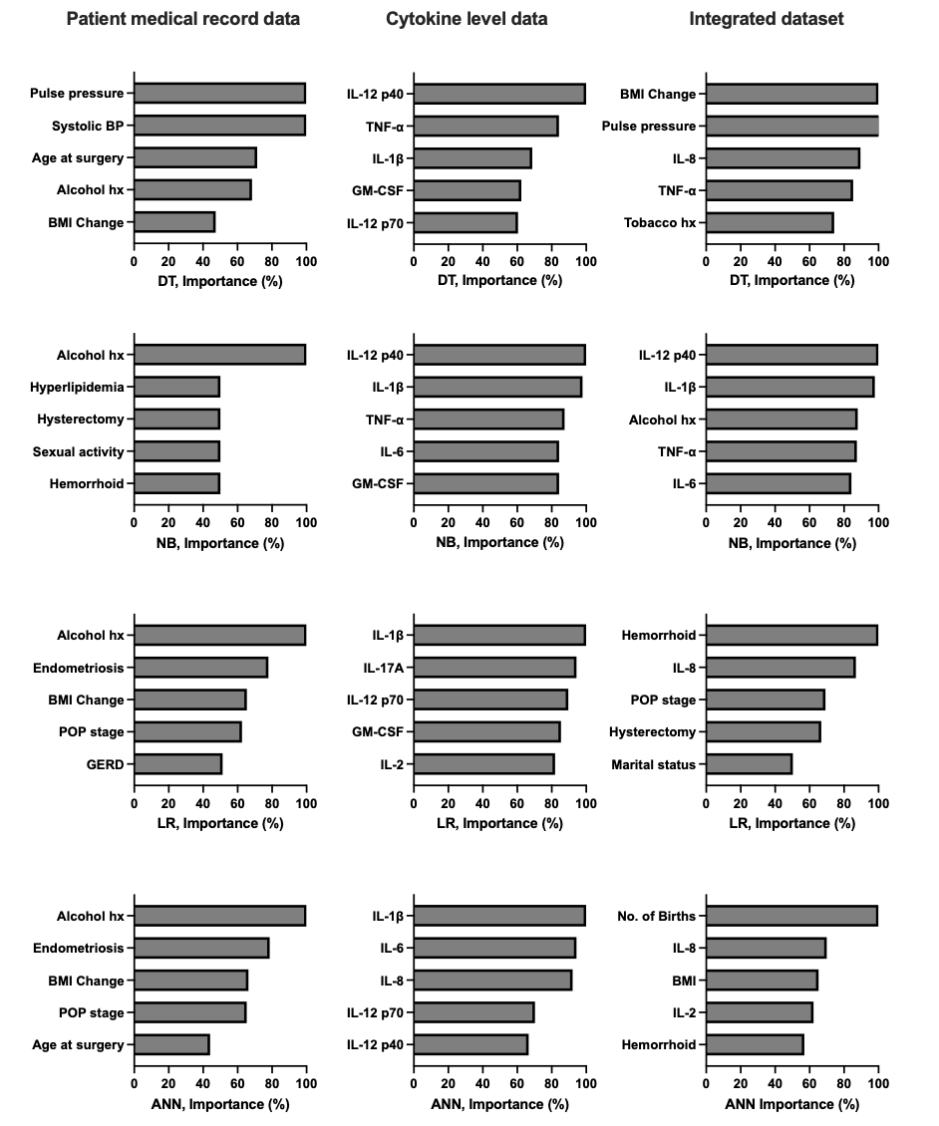
Four supervised machine learning models were used to identify important factors capable of yielding effective predictive analysis: decision tree (DT), Naïve Bayes (NB), logistic regression (LR), and artificial neural network (ANN). Each model was performed with patient medical record data (left), blood cytokine levels following incubation with surgical mesh (center), or an integrated dataset of both patient medical record data and blood cytokine levels (right). The importance of the 5 most important factors to predict mesh exposure in each model is indicated as a percentage. BP: blood pressure; GERD: gastroesophageal reflux disease; hx: history; IL: interleukin; TNF-α: tumor necrosis factor-α; GM-CSF: granulocyte-macrophage colony-stimulating factor.

Each of 4 supervised machine learning models, DT, NB, LR, and ANN, was implemented on 3 separate datasets: patient medical record data (21 variables), blood cytokine levels following incubation with surgical mesh (13 variables), and an integrated dataset of both medical data and blood cytokine levels (34 variables). The 5 most important factors that predict mesh exposure in each model are presented in Figure 1.

When the models were trained with patient medical record data alone, history of alcohol usage was the most important factor for NB, LR, and ANN models, and it also accounted for greater than 60% of the importance in the DT model. BMI change was among the 5 most relevant factors in 3 of the models, DT, LR, and ANN. In total, 3 additional factors were among the 5 most relevant factors in 2 of the 4 models: medical history of endometriosis (LR and ANN, >77% importance), POP stage (LR and ANN, >60% importance), and age at surgery (DT and ANN, >40% importance).

When models were trained with blood cytokine levels alone, IL-1β was the most important cytokine to predict mesh exposure in LR and ANN models and among the 5 most important cytokines in NB and DT models, while IL-12 p40 was the most important cytokine in DT and NB models and among the 5 most important cytokines in the ANN model. In total, 2 cytokines exhibited>60% importance in 3 models: granulocyte-macrophage colony-stimulating factor in DT, NB, and LR models and IL-12 p70 in DT, LR, and ANN models. In total, 2 cytokines exhibited>80% importance in 2 models: TNF-α in DT and NB models and IL-6 in NB and ANN models.

Finally, the predictive models were also performed on an integrated dataset of both medical record data and blood cytokine levels. IL-8 was among the 5 most important factors (>70% importance) in 3 models, DT, LR, and ANN. In total, 2 factors were among the 5 most important in 2 models: TNF-α in DT and NB models and hemorrhoids in LR and ANN models. Factors of highest importance in all 4 models included a combination of health care characteristics and blood cytokine levels. For the NB model, the 5 most important factors in the integrated dataset matched those identified to be of highest importance in the individual datasets. In the DT model, 3 factors identified to be of high importance in individual datasets were also of the highest importance in the integrated dataset, while 2 previously unidentified factors rose to high importance within the integrated dataset. In contrast, ANN and LR models identified four unique factors to be of highest importance within the integrated dataset.

Statistics were generated for the performance of each supervised machine learning model (Table 2). Each model achieved the highest prediction accuracy when using the integrated dataset, ranging from 78% (14/18) to 94% (17/18), compared to either medical record data or blood cytokine levels alone, which scored ranges of 33% (6/18) to 50% (9/18) and 50% (9/18) to 83% (15/18), respectively. Similarly, the highest training accuracy for each model was observed when using the integrated dataset, as opposed to either medical record data or blood cytokine levels alone. When comparing different models, both LR and ANN achieved the highest training and prediction accuracies with the integrated dataset. In fact, when the LR and ANN models were trained with the integrated dataset to predict mesh exposure, a training accuracy of 91% (38/42) and a prediction accuracy of 94% (17/18) were observed in combination with sensitivity and specificity ranging from 89% (16/18) to 100% (18/18).

**Table 2.** Summary of supervised machine learning model statistics. Patient medical record data (Med), blood cytokine levels following incubation with surgical mesh (Cyto), and an integrated dataset of both patient medical record data and blood cytokine levels (Int) were used to predict the presence or absence of pelvic organ prolapse (POP) postsurgical mesh exposure; 70% (42/60) of observations were used for training, and 30% (18/60) of observations were used for testing.

Model and dataset		Training accuracy, n (%)	Prediction accuracy, n (%)	95% CI		Sensitivity, n (%)	Specificity, n (%)	Prediction κ
Naïve Bayes								
	Med	16 (37)	9 (50)	0.118-0.882		0 (0)	18 (100)	0
	Cyto	26 (62)	11 (61)	0.357-0.827		6 (33)	16 (89)	0.222
	Int	31 (73)	16 (89)	0.653-0.986		14 (78)	18 (100)	0.778
Decision tree								
	Med	18 (43)	6 (33)	0.0433-0.777		12 (67)	0 (0)	0.333
	Cyto	27 (64)	11 (61)	0.57-0.827		6 (33)	16 (89)	0.222
	Int	28 (67)	14 (78)	0.524-0.936		12 (67)	16 (89)	0.556
Logistic regression								
	Med	11 (27)	6 (33)	0.043-0.777		6 (33)	6 (33)	0.333
	Cyto	31 (73)	9 (50)	0.26-0.740		10 (56)	8 (44)	0.000
	Int	38 (90)	17 (94)	0.727-0.999		18 (100)	16 (89)	0.889
Artificial neural network								
	Med	17 (40)	9 (50)	0.118-0.882		6 (33)	12 (67)	0.000
	Cyto	33 (79)	15 (83)	0.586-0.964		14 (78)	16 (89)	0.667
	Int	38 (90)	17 (94)	0.727-0.999		16 (89)	18 (100)	0.889

## Discussion

### Summary

Machine learning is gaining acceptance as a tool for predicting medical conditions, including treatment prognosis, disease progression, and surgical outcomes [7,9,11,12]. In particular, literature reports have illustrated the utility of predictive models that incorporate both health care data and biomarkers [13,14]. These successes lay the groundwork for a pioneering method that integrates patient medical records and biomarker measurements for predicting mesh-related postsurgical outcomes. Patients with POP, in particular, could benefit from such a personalized approach. These patients currently make surgical decisions based solely on risk factors derived from demographic statistics of the general population of surgical patients with POP, while individual medical history and personalized responses to the biomaterial used in the surgery could better inform their decision. Our previous study demonstrated the efficacy of predictive models that use biomaterial-induced blood cytokine-expression profiles to accurately predict mesh exposure following surgical intervention for POP using transvaginal polypropylene mesh [10]. In this exploratory study, we examined the benefit of combining continuous and categorical data from health care records and biomarker measurements to build supervised machine learning models and develop more inclusive and efficient predictive models with a higher rate of prediction accuracy, rendering such models more favorable as a novel, noninvasive, personalized clinical decision tool.

### Principal Findings and Comparison to Prior Work

Comparative statistical analysis of medical record data of patients with POP with transvaginal mesh is illustrated in Table 1. Even though this study had a small population of 20 patients, the median outcomes in mesh-exposure and nonexposure groups were comparable to those reported in other studies with a larger study cohort [15,16]. The current dataset revealed that sexually active patients were more prone to experiencing mesh exposure, a trend similarly observed by Kaufman et al [4]. This observation could be attributed to the mechanical challenges posed by sexual activity, potentially leading to friction-induced issues with the mesh implant [17]. In contrast, within the current dataset, variables such as BP, medical diagnoses, education levels, BMI, age at surgery, and parity numbers were not significant risk factors when comparing patients with or without mesh exposure. Similarly, Long et al [18] found no disparity in BMI between the 2 groups. Additionally, Chavez et al [16] noted that risk factors including obesity, diabetes, and smoking status were not associated with vaginal mesh exposure, aligning with our result.

While significant associations were not evident within many individual health care data categories, implementing supervised machine learning models on such data both reinforced groups identified via comparative statistics and revealed new groups associated with patients who experienced mesh exposure. For instance, the history of alcohol usage, indicated as important via comparative statistics, was also considered the most important factor in NB, LR, and ANN models, while BP variables were newly identified as important in the DT model. From a clinical perspective, alcohol use could contribute to malnutrition and inflammation, which may affect the healing process and influence the outcomes of mesh surgery [19]. Similarly, changes in BP and pulse pressure could indicate cardiovascular or systemic issues that may impact surgical planning or postoperative recovery [20]. However, using supervised machine learning models on patient medical record data alone yielded the lowest prediction accuracy rate when compared to models trained with either cytokine levels or an integrated dataset (Table 2). Similarly, low predictive power was reported in Taneja et al [21] when health care data alone was employed in their predictive model [21]. Thus, relying solely on medical record data to construct predictive models does not provide a comprehensive representation. Given an individual’s varying inflammatory response [10,22], as well as their race and genetic components [23,24], merely identifying commonalities among medical record data may not accurately represent predictors of the mesh exposure postsurgery.

Previous studies have indicated a relationship between elevated levels of pro-inflammatory cytokines and postsurgical complications [25-27]. When creating predictive models trained with 13 biomaterial-induced cytokines known to influence wound healing, the models predicted with a higher prediction accuracy of 50% (9/18) to 83% (15/18) compared to models trained with patient medical record data (Table 2). These supervised machine learning models demonstrated that IL-1β was one of the most important cytokines to predict mesh exposure across all four models. Elevated levels of IL-1β have been associated with various diseases, including obesity, cardiovascular diseases, cancer, and periodontitis [28-30]. Moreover, IL-1β was increased in a murine model following hernia polypropylene mesh implantation [31], mirroring our findings. Additionally, IL-12 p40 played a pivotal role in DT and NB models. This pro-inflammatory cytokine, which serves as a critical link between the innate and adaptive immune response, is observed in inflammatory conditions, such as pulmonary sarcoidosis and inflammatory bowel diseases [32-34].

All 4 supervised machine learning models achieved their highest prediction accuracies when trained with an integrated dataset comprising both patient medical record data and biomaterial-induced blood cytokine levels. This integrated dataset proved particularly effective when used in LR and ANN models, achieving 94% (17/18) prediction accuracy. The approach also exceeds predictive health care models for other surgical outcomes that used only cytokine-based [35] or electronic health record-based approaches [36]. In parallel, Taneja et al [21] demonstrated that their sepsis predictive models had higher predictive power when combined data were used, rather than either electronic medical record or biomarker data alone. Interestingly, this integrated approach also identifies new factors of high importance. In contrast to the models trained on cytokine expression alone, models trained with integrated data observed that IL-8 was among the important predictors in DT, LR, and ANN models, and TNF-α in DT and NB models. IL-8 and TNF-α are 2 prominent pro-inflammatory cytokines elevated in patients postsurgery, including mesh implantation and pelvic surgery [37,38]. In contrast to the models trained on medical record data alone, hemorrhoids were considered one of the important factors in LR and ANN models trained with the integrated dataset. Models trained with the integrated dataset also outperformed those trained with either dataset alone in terms of training accuracy, sensitivity, and specificity across all four models (Table 2).

### Limitations and Future Directions

Despite employing rigorous research methodologies, it is imperative to acknowledge certain limitations inherent in this study. As a pilot study, the population size was limited to 20 patients within a single hospital system. Even though it was on a small scale, this study yielded a prediction accuracy as high as 94% (17/18), surpassing comparable predictive models that ranged from 71% to 86% in prediction accuracy across a population size of 198-467 [35,39], thus affirming the validity of our approach. Future research will involve a larger participant pool drawn from multiple hospitals to further confirm these results. To mitigate potential confounders, patients with POP recurrence or taking medication that would alter inflammatory response were excluded. Future studies with a larger population will also enable matching participants with respect to these and other potentially confounding variables. In the analysis of the integrated dataset, medical record data were repeated 3 times to match the structure of the cytokine dataset, which contained 3 repeated measurements. This approach was employed to balance the contribution of both data types. This may represent to a potential limitation, although the resulting variable importance graphs for the integrated dataset demonstrated a relatively equal contribution from both the cytokine and medical record data, mitigating concerns that the repetition of the medical record data led to overfitting. Despite these limitations, the results of this pilot study highlight the importance of integrating inflammatory biomarkers with medical record data in the prediction of postsurgical mesh exposure.

### Conclusions

Supervised machine learning models demonstrated significantly higher prediction accuracy when trained with an integrated dataset comprising both patient medical record data and biomaterial-induced blood cytokine levels, surpassing the performance of models trained using either dataset alone. These results demonstrate for the first time the advantage of an approach integrating medical record data and blood biomarkers. This innovative approach to predicting surgical outcomes following POP repair via transvaginal insertion of polypropylene mesh holds promise for both patients and surgeons. Additionally, such a method could be applied to the study of other mesh-related surgeries, such as those used to repair POP transabdominally or to treat hernia and stress urinary incontinence. By presenting a more personalized decision-making tool, this approach offers a more accurate depiction of potential outcomes, thereby enhancing informed decision-making and improving patient care.

## Abbreviations

ANN: artificial neural network
BP: blood pressure
DT: decision tree
IL: interleukin
LR: logistic regression
NB: Naïve Bayes
POP: pelvic organ prolapse
TNF-α: tumor necrosis factor alpha

## References

[1] Hullfish KL, Bovbjerg VE, Gurka MJ, Steers WD (2008). Surgical versus nonsurgical treatment of women with pelvic floor dysfunction: patient centered goals at 1 year. J Urol.

[2] Seifalian A, Basma Z, Digesu A, Khullar V (2023). Polypropylene pelvic mesh: what went wrong and what will be of the future?. Biomedicines.

[3] Syed KK, Consolo MJ, Gousse AE, Repair A (2021). Anterior vaginal wall prolapse repair and the rise and fall of transvaginal mesh. Did we come full circle? A historical perspective. Urology.

[4] Kaufman Y, Singh SS, Alturki H, Lam A (2011). Age and sexual activity are risk factors for mesh exposure following transvaginal mesh repair. Int Urogynecol J.

[5] Akyol A, Akca A, Ulker V (2014). Additional surgical risk factors and patient characteristics for mesh erosion after abdominal sacrocolpopexy. J Obstet Gynaecol Res.

[6] Artsen AM, Liang R, Meyn L, Bradley MS, Moalli PA (2023). Dysregulated wound healing in the pathogenesis of urogynecologic mesh complications. Sci Rep.

[7] Jung S, Park K, Ihn K (2022). Predicting graft failure in pediatric liver transplantation based on early biomarkers using machine learning models. Sci Rep.

[8] Chowdhury NI, Li P, Chandra RK, Turner JH (2020). Baseline mucus cytokines predict 22-item sino-nasal outcome test results after endoscopic sinus surgery. Int Forum Allergy Rhinol.

[9] Kawakita S, Beaumont JL, Jucaud V, Everly MJ (2020). Personalized prediction of delayed graft function for recipients of deceased donor kidney transplants with machine learning. Sci Rep.

[10] Waugh ML, Boltin N, Wolf L (2023). Prediction of pelvic organ prolapse postsurgical outcome using biomaterial-induced blood cytokine levels: machine learning approach. JMIR Perioper Med.

[11] Li B, Shaikh F, Zamzam A, Syed MH, Abdin R, Qadura M (2024). A machine learning algorithm for peripheral artery disease prognosis using biomarker data. iScience.

[12] Thorwarth RM, Scott DW, Lal D, Marino MJ (2021). Machine learning of biomarkers and clinical observation to predict eosinophilic chronic rhinosinusitis: a pilot study. Int Forum Allergy Rhinol.

[13] Reddy BK, Delen D, Agrawal RK (2019). Predicting and explaining inflammation in Crohn’s disease patients using predictive analytics methods and electronic medical record data. Health Informatics J.

[14] Ryan CT, Zeng Z, Chatterjee S (2023). Machine learning for dynamic and early prediction of acute kidney injury after cardiac surgery. J Thorac Cardiovasc Surg.

[15] Niu K, Lu YX, Shen WJ, Zhang YH, Wang WY (2016). Risk factors for mesh exposure after transvaginal mesh surgery. Chin Med J (Engl).

[16] El-Khawand D, Wehbe SA, O’Hare PGI, Arunachalam D, Vakili B (2014). Risk factors for vaginal mesh exposure after mesh-augmented anterior repair. Female Pelvic Med Reconstr Surg.

[17] Kowalik CR, Lakeman MME, de Kraker AT, Roovers JPWR (2019). Effects of mesh-related complications in vaginal surgery on quality of life. Int Urogynecol J.

[18] Long CY, Lo TS, Wang CL, Wu CH, Liu CM, Su JH (2012). Risk factors of surgical failure following transvaginal mesh repair for the treatment of pelvic organ prolapse. Eur J Obstet Gynecol Reprod Biol.

[19] Daher GS, Choi KY, Wells JW, Goyal N (2022). A systematic review of oral nutritional supplement and wound healing. Ann Otol Rhinol Laryngol.

[20] Mogoanta SS, Paitici S, Mogoanta CA, Zaghal A, Rifai AE (2021). Abdom Surg.

[21] Taneja I, Reddy B, Damhorst G (2017). Combining biomarkers with EMR data to identify patients in different phases of sepsis. Sci Rep.

[22] Pos Z, Selleri S, Spivey TL (2010). Genomic scale analysis of racial impact on response to IFN-alpha. Proc Natl Acad Sci U S A.

[23] Hoffmann SC, Stanley EM, Cox ED (2002). Ethnicity greatly influences cytokine gene polymorphism distribution. Am J Transplant.

[24] Gökçe İ (2021). Effects of human genetic factors (ethnicity and race) on clinical severity of SARS-CoV-2 (COVID-19). JEB Med Sci.

[25] Mathe Z, Serban RC, Pintilie I, Somkereki C, Hutanu A, Scridon A (2018). Postoperative interleukin-8 levels are related to the duration of coronary artery bypass grafting surgery and predict in-hospital postsurgical complications. Rev Rom Med Lab.

[26] Paruk F, Chausse JM (2019). Monitoring the post surgery inflammatory host response. J Emerg Crit Care Med.

[27] Tsilimigras DI, Sigala F, Karaolanis G (2018). Cytokines as biomarkers of inflammatory response after open versus endovascular repair of abdominal aortic aneurysms: a systematic review. Acta Pharmacol Sin.

[28] Khafagy R, Dash S (2021). Obesity and cardiovascular disease: the emerging role of inflammation. Front Cardiovasc Med.

[29] Pani P, Tsilioni I, McGlennen R (2021). IL-1B(3954) polymorphism and red complex bacteria increase IL-1β (GCF) levels in periodontitis. J Periodontal Res.

[30] Tulotta C, Ottewell P (2018). The role of IL-1B in breast cancer bone metastasis. Endocr Relat Cancer.

[31] Heymann F, von Trotha KT, Preisinger C (2019). Polypropylene mesh implantation for hernia repair causes myeloid cell-driven persistent inflammation. JCI Insight.

[32] Abdi K (2002). IL-12: the role of p40 versus p75. Scand J Immunol.

[33] Shigehara K, Shijubo N, Ohmichi M (2003). Increased circulating interleukin-12 (IL-12) p40 in pulmonary sarcoidosis. Clin Exp Immunol.

[34] Verstockt B, Salas A, Sands BE (2023). IL-12 and IL-23 pathway inhibition in inflammatory bowel disease. Nat Rev Gastroenterol Hepatol.

[35] Chen Z, Chen L, Yao G, Yang W, Yang K, Xiong C (2020). Novel blood cytokine-based model for predicting severe acute kidney injury and poor outcomes after cardiac surgery. J Am Heart Assoc.

[36] Weller GB, Lovely J, Larson DW, Earnshaw BA, Huebner M (2018). Leveraging electronic health records for predictive modeling of post-surgical complications. Stat Methods Med Res.

[37] Parpoudi S, Mantzoros I, Gkiouliava A (2022). Effect of N-acetyl-L-cysteine on inflammation after intraperitoneal mesh placement in a potentially contaminated environment: an experimental study in the rat. Asian J Surg.

[38] Shi JY, Bergerat-Thompson AM, Mitchell CM (2023). The impact of the vaginal microbiome on vaginal epithelial wound healing. Research Square.

[39] Chu CS, Lee NP, Adeoye J, Thomson P, Choi SW (2020). Machine learning and treatment outcome prediction for oral cancer. J Oral Pathol Med.

[40] IntegratedDataset. GitHub.

